# Greater risk-taking by non-native than native shrimp: an advantage in a human-disturbed environment?

**DOI:** 10.1186/s12862-024-02330-2

**Published:** 2024-11-19

**Authors:** Alfredo Escanciano Gómez, Charlotte Ipenburg, Ulrika Candolin

**Affiliations:** Organismal and Evolutionary Biology Research Programme, University of Helsinki, Helsinki, Finland

**Keywords:** Anthropogenic change, Behaviour, Environmental change, Habitat, Invasive, Nonindigenous, Optimality, Prawn, Predation risk, Vegetation

## Abstract

**Background:**

The invasion of non-native species into ecosystems is a growing human-induced problem. To control their spread and population growth, knowledge is needed on the factors that facilitate or impede their invasions. In animals, traits often associated with invasion success are high activity, boldness, and aggression. However, these traits also make individuals susceptible to predation, which could curb population growth. We investigated if a recent invader into the Baltic Sea, the shrimp *Palaemon elegans*, differs in risk-taking from a native shrimp, *P. adspersus*. We recorded activity, habitat choice, and response to perceived predation threat of both species.

**Results:**

We found the invading shrimp to take greater risks than the native one; while the native shrimp adjusted its behaviour to habitat structure and exposure to a perceived predator, the non-native shrimp did not, and it resumed normal activity sooner after a perceived predation threat. Despite the greater risk taking by the non-native shrimp, its population has grown rapidly during the last two decades in the investigated area and is now larger than that of the native shrimp.

**Conclusions:**

We discuss plausible explanations for the population growth of the invader, including the recent decline in predatory fishes that could have reduced the cost of risk-taking, and anthropogenic eutrophication that has increased food abundance could have allowed the population growth. These results stress the need to assess the optimality of the behaviours of both native and non-native species when investigating the factors that influence invasion success in human-disturbed environments.

**Supplementary Information:**

The online version contains supplementary material available at 10.1186/s12862-024-02330-2.

## Background

The invasion of non-native species into ecosystems is a growing ecological problem, caused by the increased mobility of humans and the destruction of natural habitats [1, 2]. Characteristics of animals that often make them successful in invading new areas are high activity, boldness and aggression [3–7]. These traits frequently give invaders an advantage over native species in the competition for resources and facilitate invasion success [8, 9]. This indicates that superiority in interspecific competition can act as a driver of invasion spread. However, traits such as high activity and boldness also make invaders more susceptible to predation, which can reduce their survival probability and limit their population growth in the invaded area [10, 11]. This can lead to a trade-off situation between competitive ability and predation risk, in which case optimal behaviour may depend on the structure of the habitat and the visibility to predators [12–14].

Trade-offs between competitive ability and predation risk has been investigated within communities of native species [15–17]. However, it has surprisingly seldom been considered in the framework of species invasion success. Yet, invasive species often go through a boom-bust dynamics where initial population growth is followed by a dramatic decline in abundance. This is often due to predators learning to prey on the invaders, which the regulates their population growth [18–20]. However, the regulation could be weaker in human-disturbed environments if the disturbance reduces the competitive ability and viability of native species and, in so doing, give invaders an advantage in the competition for resources, which could facilitate their invasion success [18].

Common invaders in shallow aquatic ecosystems are shrimps of the family Palaemonidae (Crustacea, Decapoda). They have spread around the world and are influencing the behaviour and population dynamics of native species, with further consequences for ecosystem structure and function [21–24]. In the Baltic Sea, the shrimp *Palaemon elegans* recently invaded the coast of Southern Finland [25]; it was first detected in 2003 [26], after which the population rapidly grew and is today larger than that of the native shrimp *P. adspersus* [25]. *P. elegans* originates from the Mediterranean or the Black Sea, but whether it has spread from more southern populations in the Baltic Sea or represents a separate introduction is unknown [27].

A factor that could have contributed to the invasion success of the non-native shrimp is its high activity level and boldness [25, 28–32]. This could give it an upper hand in the competition for space and food with native species. Studies on the non-native shrimp in the Baltic Sea have found it to coexist with a native competitor for space and food, the threespine stickleback (*Gasterosteus aculeatus*), a mesopredatory fish, by adjusting its microhabitat choice and activity to the density of the fish [28, 33]. However, behavioural interactions with the closely related native shrimp, which uses the same ecological niche as the invader is unknown [30]. The Baltic Sea is currently undergoing large environmental changes in both abiotic and biotic factors because of human activities, such as climate change, the overharvesting of top-predators and increased primary production because of eutrophication [34, 35]. These changes are influencing the behaviour and population dynamics of native species [36–38]. Similarly, the response of the non-native shrimp to predation risk in the disturbed Baltic Sea environment is unknown. Shrimps are common prey of local predatory fishes in the Baltic Sea, such as perch *Perca fluviatilis*, Northern pike *Esox lucius*, and cod *Gadus morhua*, and high activity could increase exposure to these [39–42]. Thus, while the high activity level of the non-native shrimp could improve its success in the competition for resources, it may simultaneously increase predation risk. Whether the shrimp can reduce its susceptibility to predators by adjusting its activity to habitat structure and visibility to predators is unknown. Vegetation shelters shrimps against predators and a careful adjustment of habitat choice and activity to habitat structure could reduce predation risk [43–45].

Based on the sheltering function of vegetation, we hypothesized that the non-native shrimp would show (i) a stronger preference for vegetated habitats than the native shrimp given its higher activity level that increases its exposure to predators, (ii) a stronger adjustment of behaviour to habitat structure when no choice among habitats is possible (i.e., be less active in more open habitats) in order to reduce its predation risk, and (iii) a stronger anti-predator response to the appearance of a predator because of its higher activity and exposure to predators. To assess these hypotheses, we performed a series of experiments that investigated if the two species differ in (i) choice between an open and a vegetated habitat, (ii) adjustment of behaviour to habitat structure (general activity, aggressive interactions, and feeding activity) when no choice among habitats is possible, and (iii) response to a perceived predation threat, a model of a Northern pike.

## Methods

### Collection and housing

We caught *P. elegans* and *P. adspersus* from a rocky shore close to Tvärminne Zoological station in Southern Finland using Plexiglas traps [46]. They were caught in early summer after they had arrived from deeper water. The habitat contains both bare stones and those overgrown by the filamentous algae *Cladophora glomerata* and bladder wrack *Fucus vesiculosus*. We transported the shrimps, about 300 individuals of each species, to the University of Helsinki and housed them in several holding tanks (120 l) filled with water of the same salinity as at the collection site (5 ppm), species separated, with 20 individuals per tank. The tanks were aerated with air stones and kept at 15 °C and a 18:6 light cycle to simulate natural conditions. Stones with filamentous algae were distributed over the bottom to create a patchily vegetated habitat. We fed the shrimp defrosted chironomid larvae once a day, ad libitum, to ensure that all individuals had the same saturation during the experiments. We allowed them to acclimate to the housing conditions for at least 2 days before using them in the experiments. In the trials, we combined individuals from different holding tanks to eliminate the possibility of prior experience, a tank effect, confounding the results. Both species are active during the day [29, 31] and all experiments were conducted during their active time, between 9 and 15. Light and temperature conditions followed that in holding tanks. No individuals were reused within or among the three experiments.

### Experiment 1. Habitat choice

We created two equal sized habitats (30 × 50 cm, water depth 20 cm) in large tanks (30 × 100 cm): open and vegetated. The open habitat contained only bare stones, 3–8 cm in diameter, while the vegetated contained bunches of artificial vegetation attached to the stones, 15-cm long, thin, green polypropylene strings that mimicked filamentous algae and covered about 80% of the bottom [47]. The tanks were visually separated from other tanks, the surroundings, and the observers by using curtains. We added groups of 10 adult individuals of the same species to each tank and left them for 24 h to acclimate to the new conditions. After acclimation, we filmed the individuals for 10 min for later analyses of habitat choice. We performed 8 replicates, separately for each species, using different individuals in each replicate.

### Experiment 2. Adjustment of behaviour to habitat structure

To assess if individuals adjust their behaviour to the habitat when no choice among habitats is possible, we repeated the procedures described for habitat choice but with the tanks (30 × 100 cm) containing only an open or a vegetated habitat. After the 24 h of acclimatisation, we added five defrosted chironomid larvae to the middle of the tank to assess behaviour and feeding activity of the shrimps. We performed 8 replicates, separately for each species, using different individuals in each replicate.

### Experiment 3. Responses to predation threat

Given that shrimps often move in smaller groups, we placed two individuals of the same species into a tank (30 × 100 cm, water depth 20 cm) with rows of artificial vegetations along both short ends (see description of artificial vegetation in [48]) and 60 cm of open habitat between the rows. After 24 h of acclimatisation, we added defrosted chironomid larvae to the middle of the open habitat. When both shrimp were feeding on the chironomids in the open habitat, we allowed a model of a pike to slowly appear on the frontside of the tank. We manually moved the pike using transparent rods, and withdraw it after 10 s of exposure. We performed 8 replicates, separately for each species, using different individuals in each replicate.

### Behavioural analyses

Behaviour was recorded by AEG using the software Boris [49]. In the habitat choice experiment (experiment 1), we recorded the number of individuals in each habitat every one minute, resulting in 10 observations per replicate. We calculated the average of these 10 observations.

In the behavioural adjustment experiment (experiment 2), we recorded activity throughout the 10 min of filming; we defined general activity as the number of individuals that crossed the middle of the aquarium, aggression as the number of times an individual attacked or chased another individual, and feeding activity as the number of individuals feeding every one minute.

In the predation threat experiment (experiment 3), we recorded the latency of the shrimp response to the model pike, the form of the response (freezing within the open habitat for at least 5 s or moving to one of the vegetated habitats), and the latency of the shrimp to resume their moving activity within the open habitat.

### Statistical analyses

We analysed the data using the software IBM SPSS Statistics v 29. We tested habitat preferences of each species with one sample t-tests and compared habitat choice, activity, aggression and feeding behaviour of the two species with linear models, defining species and habitat as fixed factors. To compare the latency of the response to perceived predation threat, we used mixed models with species as fixed factor and pair as random factor (as the two individuals were dependent within replicates). We checked the assumptions of the analyses - heteroscedasticity and normal distribution of residuals – using histograms and Q-Q plots.

## Results

**Fig. 1:**
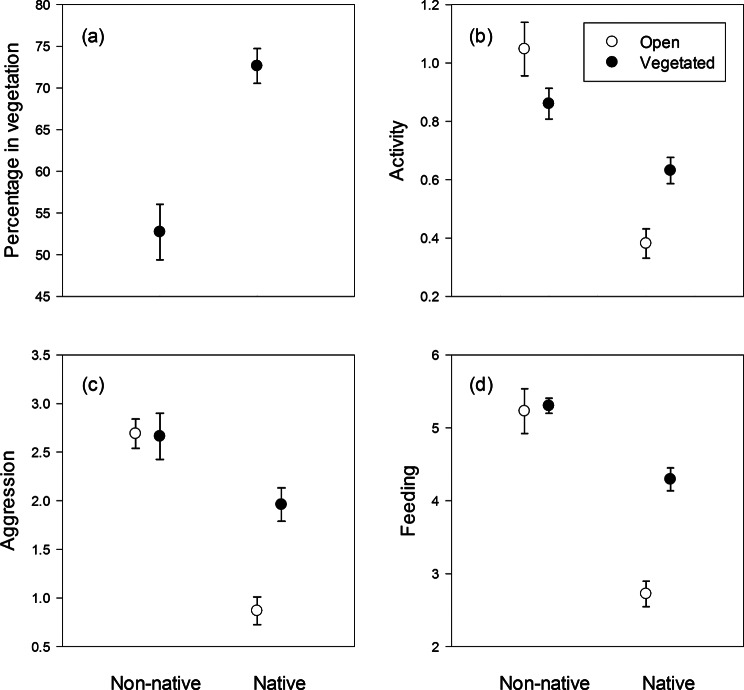
Behaviour of ten individuals of the non-native *P. elegans* or the native *P. adspersus* during 10 min. Data shows (**a**) percentage of individuals in the vegetated habitat in experiment 1, and (**b**) number of individuals crossing the middle of the tank per minute, (**c**) number of aggressive interactions per minute, and (**d**) number of individuals feeding per minute in the two habitats in experiment 2. Values are averages ± SE, *N* = 8 per treatment (with each replicate including observations of ten individuals)

In experiment 1, the non-native shrimp *P. elegans* differed in habitat choice from the native shrimp *P. adspersus* (F_1,14_ = 25.6, *P* < 0.001, Fig. 1a,: which showed no clear preference for either habitat (one-sample t-test of deviation from 0.5, t_7_ = 0.81, *P* = 0.45), while the native shrimp preferred the vegetated habitat (t_7_ = 10.85, *P* < 0.001).

In experiment 2, the non-native shrimp adjusted its activity less to habitat structure than the native shrimp, and it was more active than the native shrimp for all recorded behaviours, general activity, aggression, and feeding (Table 1; Fig. 1b-d). Post-hoc tests show that the non-native shrimp showed a weak tendency to reduce its activity in the vegetated habitat, while the native shrimp reduced its activity in the open habitat (Table 2; Fig. 1b). The non-native shrimp did not adjust aggression or feeding activity to habitat structure, while the native shrimp reduced these activities in the open habitat (Table 2; Fig. 1c-d).

**Fig. 2:**
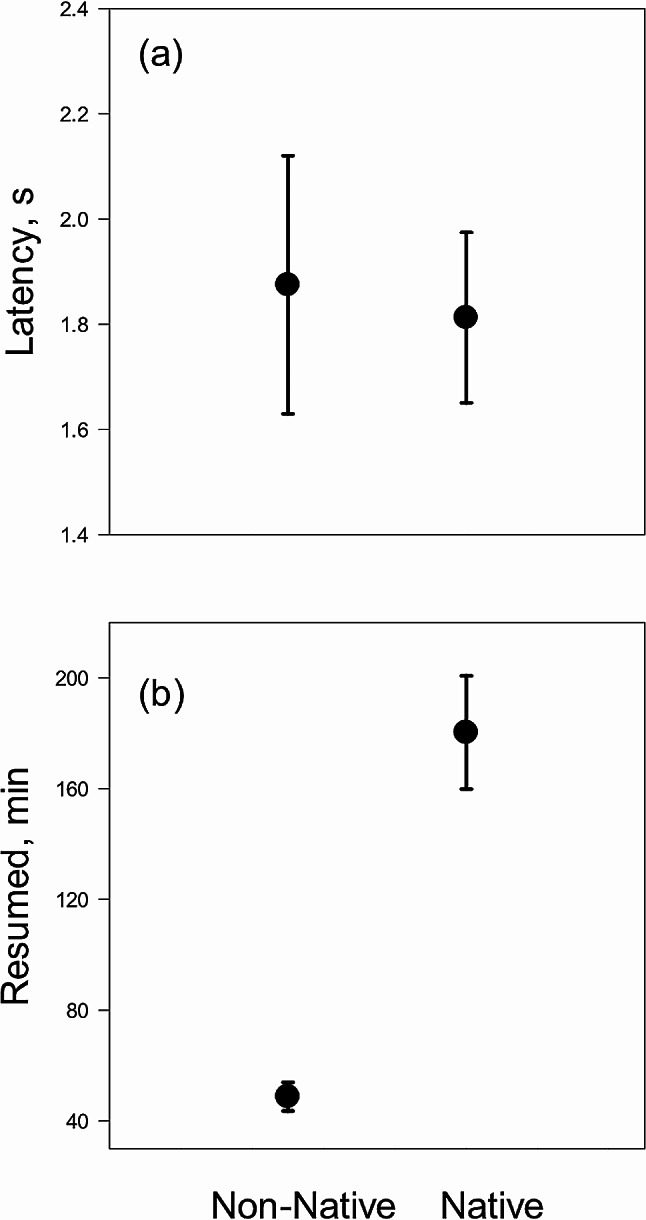
Behavioural responses of two individuals of the non-native *P. elegans* or the native *P. adspersus* to a perceived predation threat, the appearance of a model pike in experiment 3. Data shows (**a**) latency to respond to the threat, and (**b**) time until resuming normal behaviour. Values are averages ± SE, *N* = 8 for each species (with each replicate including observation of two individuals)

In experiment 3, the two species did not differ in latency to responding to the model pike (mixed model with species as fixed factor and individual as random factor: F_1,14_ = 0.45, *P* = 0.835, Fig. 2a). All individuals responded by seeking shelter in the vegetation and staying motionless. The non-native shrimp left the shelter sooner than the native shrimp (F_1,14_ = 38.90, *P* < 0.001, Fig. 2b).

**Table 1:** Influence of species (non-native *P. Elegans* or native *P. adspersus*) and habitat (open or vegetated) on the behaviour of 10 individuals during 10 min in experiment 2. Significant results are marked in bold

	Activity		Aggression		Feeding	
	F_1,28_	P	F_1,28_	P	F_1,28_	P
Species	50.93	**< 0.001**	49.40	**< 0.001**	77,34	**< 0.001**
Habitat	0.25	0.622	8.89	**0.006**	16.92	**< 0.001**
Species*Habitat	12.15	**0.002**	9.71	**0.004**	14.05	**0.001**

Data was analysed using linear models with species and habitat as fixed factors

**Table 2:** Influence of habitat (open or vegetated) on the behaviour of 10 individuals during 10 min, measured for the non-native *P. Elegans* and the native *P. adspersus* in experiment 2. Significant results are marked in bold

		F_1,14_	*P*
*P. elegans*	Activity	3.14	0.098
	Aggression	0.01	0.925
	Feeding	0.051	0.825
*P. adspersus*	Activity	13.70	**0.002**
	Aggression	23.87	**< 0.001**
	Feeding	44.8	**< 0.001**

Data was analysed using linear models with habitat as fixed factor

## Discussion

The non-native shrimp *P. elegans*, which was first detected along the coast of Southern Finland in 2003 [26], was more active than the native shrimp *P. adspersus* for all recorded behaviours (swimming activity, aggression, and feeding) irrespective of habitat. This agrees with earlier research, which has found the non-native shrimp to be more active than the native species [25, 28–31]. Contrary to our expectations, it did not show a stronger preference for the vegetated habitat than the native shrimp. Instead it showed no habitat preference when offered a choice between a vegetated and an open habitat, although more vegetated habitats offer better shelter for shrimps against predators [43–45], and the native shrimp strongly preferred the vegetated habitat. The non-native shrimp did not adjust its activity to habitat structure when no choice among habitats was possible, while the native shrimp did, and it resumed normal activity sooner after a predation threat than the native shrimp. Thus, the responses of the non-native shrimp differed from that of the native shrimp, which preferred the vegetated habitat over the open one, strongly adjusted its behaviour to habitat structure by being less active in the open habitat, and was slower in resuming normal activity after a predation threat. These results indicate that the non-native shrimp takes larger risks than the native one.

Despite the disposition for greater risk taking in the non-native shrimp, its abundance has increased drastically along the Southern coast of Finland during the last decade [25]. Thus, its high activity and risk taking appear not to incur a fitness cost that could limit its population growth. Instead, the behaviours could have benefitted the invader in the competition for resources [29, 30]. The abundance of larger predatory fishes has declined during the last decades because of overfishing [35], which could have decreased the predation-risk cost of high activity. At the same time, the abundance of food has increased because of anthropogenic eutrophication [34], which could have allowed energetically expensive behaviours, such as high activity, as well as promoted the population growth of the invading shrimp. Shrimps feed on both animals and algae and can benefit from eutrophication and enhanced algae growth [50–52]. Thus, human-induced changes to the environment could have reduced the importance of efficient anti-predator behaviours, while providing an abundant supply of food for maintaining high activity and promoting population growth. Why, then, does the native shrimp not employ similar high activity and risk taking? This could be related to past environmental conditions, before the arrival of the non-native shrimp, when competition for resources – food and habitat – may have been weaker and high activity and boldness not needed.

In addition, the wider ecological niche of the non-native shrimp is likely to have favoured its invasion success. It has greater tolerance to extreme environmental conditions than the native shrimp and can withstand lower oxygen and salinity levels [29, 53, 54]. Thus, it could have been a stronger competitor for resources during the last decade when salinity has decreased and temperature has increased because of climate change [55]. Similarly, the non-native shrimp may have been better at withstanding the expansion of areas with low oxygen levels caused by anthropogenic eutrophication and the associated increase in decaying organic material [34, 56].

Successfully invading species are often highly active and bold, which is frequently assumed to explain their invasion success, given that it can provide an advantage in the competition for resources [3–7]. However, such behaviours could also promote population decline after a lag phase, if local predators learn to feed on the invaders, or the amount of resources needed to maintain high activity and population growth is depleted [57, 58], resulting in boom-bust dynamics [18] or S-shaped impact curve with a lag phase followed by an exponential growth phase until saturation is reached [59]. However, environmental changes that alter the costs and benefits of boldness and high activity could alter the optimal expression of these behaviours and influence population trajectories. Yet, the influence that human-induced environmental changes have on optimal behaviour and thereby on invasion success has so far received little attention.

## Conclusions

Our study shows that the non-native shrimp *P. elegans* is more active and takes larger risks than the native shrimp *P. adspersus* in a recently invaded area in the Baltic Sea. This could have benefitted the non-native shrimp in the competition for resources while at the same time incurring low costs, as the abundance of predatory fishes has been low during the time of invasion while the abundance of food has been high, because of human activities. In general, little is known about the impact of human-induced environmental changes on the costs and benefits of behaviours such as boldness and activity, and how changes in optimal behaviour can influence invasion success. Considering the rate and extent at which humans are disturbing environments, more attention should be directed to the impact of environmental change on optimal behaviour and how this influences invasion success.

## Electronic supplementary material

Below is the link to the electronic supplementary material.

Supplementary Material 1

## Data Availability

Data is provided within the supplementary information.
